# Construction and Characterization of TiN/Si_3_N_4_ Composite Insulation Layer in TiN/Si_3_N_4_/Ni_80_Cr_20_ Thin Film Cutting Force Sensor

**DOI:** 10.3390/mi12121476

**Published:** 2021-11-29

**Authors:** Ruyuan Ma, Wenge Wu, Zhenyu He, Yunping Cheng, Lijuan Liu, Yongjuan Zhao

**Affiliations:** School of Mechanical Engineering, North University of China, Taiyuan 030051, China; mry2863@163.com (R.M.); hezhenyu0229@163.com (Z.H.); ypchengbk@163.com (Y.C.); liulijuan@nuc.edu.cn (L.L.); zyj@nuc.edu.cn (Y.Z.)

**Keywords:** TiN/Si_3_N_4_ film, surface microstructure, phase composition, peak intensity, film thickness, mechanical properties

## Abstract

The measurement of cutting force is an effective method for machining condition monitoring in intelligent manufacturing. Titanium nitride films and silicon nitride films were prepared on 304 stainless steel substrates by DC-reactive magnetron sputtering and plasma-enhanced chemical vapor deposition (PECVD). The effects of substrate negative bias and nitrogen flow on the surface microstructures of TiN film were investigated. The smoothness of the film is optimal when the bias voltage is −60 V. X-ray diffraction (XRD) analysis was performed on the samples with the optimal smoothness, and it was found that when the nitrogen flow rate was higher than 2 sccm, the titanium nitride film had a mixed phase of TiN(111) and (200). It is further revealed that the change of peak intensity of TiN(200) can be enhanced by nitrogen flow. Through atomic force microscopy (AFM), it is found that the stronger the intensity of the TiN (200) peak, the smoother the surface of the film is. Finally, the effect of different film thicknesses on the hardness and toughness of the TiN/Si_3_N_4_ film system was studied by nanoindentation experiments. The nanohardness (H) of the TiN/Si_3_N_4_ film can reach 39.2 GPa, the elastic modulus (E) is 480.4 GPa, the optimal toughness value (H^3^/E^2^) is 0.261 GPa, and the sample has good insulation performance. Linear fitting of the film’s toughness to nanohardness shows that TiN/Si_3_N_4_ films with higher hardness usually have a higher H^3^/E^2^ ratio.

## 1. Introduction

**Figure 1:**
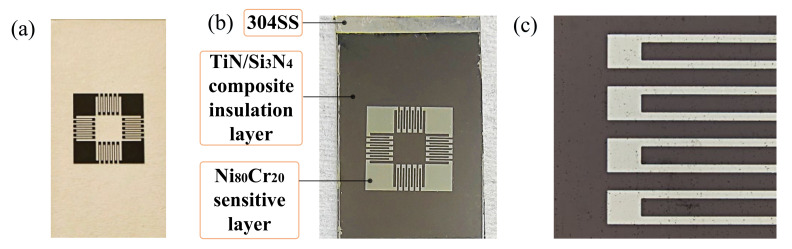
(**a**) The four thin-film resistor grids are connected into “Ring-shaped” Wheatstone bridge circuits. (**b**) Thin-film force sensor. (**c**) Partially enlarged image of resistance grid.

Measuring cutting force is an effective method for machining condition monitoring in intelligent manufacturing and can provide data support for cutting fault prediction, tool life research, and cutting parameter optimization [1,2,3]. A thin-film strain cutting force sensor is widely used because of its high precision and reliability [4,5,6,7]. Veprek et al. proposed the concept of crystalline–amorphous nanocomposite coating by modifying transition metal (TM) nitride [8,9,10,11], in which the most typical composite structure is the nc-TiN/α-Si_3_N_4_ thin-film system. Although much research has been devoted to studying the microstructure and tribological behavior of TiN/Si_3_N_4_ thin films, few studies have been carried out on their application in the field of cutting force sensors. If the film system is applied to the cutting process of high-strain rate-impact, the all-around performance of hardness and toughness becomes an important index, which is essential for improving the measurement accuracy and service life of the sensor. A thin-film force sensor with TiN/Si_3_N_4_ thin-film system has been successfully manufactured, composed of 304 Stainless-Steel substrate, TiN/Si_3_N_4_ composite insulation layer, and Ni_80_Cr_20_ sensitive layer, as shown in Figure 1:

In the TiN/Si_3_N_4_ film system, the phase composition of TiN is the key to achieving ultrahardness [12,13,14], and its interface fracture toughness primarily depends on the preferred orientation of the TiN layer, rendering the quality of the TiN layer particularly important [15,16,17]. In this paper, a TiN/Si_3_N_4_ functional film system for cutting force measurement is established, and the relationship between film thickness, nanohardness (H), elastic modulus (E), and film toughness (H^3^/E^2^) is studied in detail. This work makes preparations for future data acquisition.

## 2. Experimental Theory and Method

### 2.1. Mismatch Strain Analysis of Films by Stoney’s Classic Film Stress Formula

**Figure 2:**
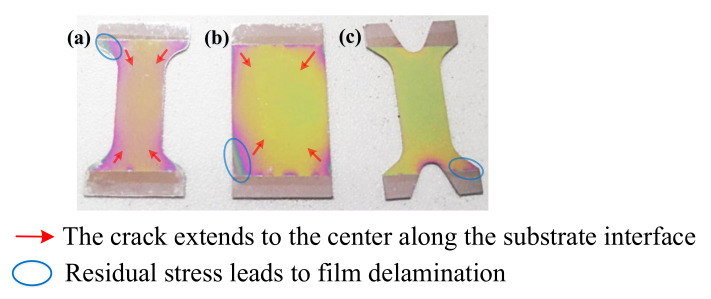
(**a**–**c**) Crack propagation in TiN/Si_3_N_4_ film system on 304SS substrates with different shapes.

Excessive mismatch strain results in extremely high residual stress in the film, which significantly affects the adhesion and fracture toughness of the film [18,19]. In J.W. Hutchinson’s theory of interface toughness [20], when the internal stress of the film is too large, the cracks extend preferentially along the substrate interface, which aligns with the experimental data we have obtained, shown in Figure 2.

The same mode of crack propagation was found on 304SS substrates with different shapes of a–b. All cracks extend from the substrate interface to the center, and some parts of the film at the substrate interface show severe delamination. It is essential to control the internal stress of the film to ensure its optimal performance.

Stress in the thin film is expressed by the Stoney equation [21,22,23]:

(1) $$\sigma_{f}=\frac{E_{0}t_{0}^{2}}{6\;\left(1-v_{0}\right)t_{i}}\Delta k\;\;\;\;\Delta k=k_{i}-k_{i-1}\;\left(i=1\ldots n\right)$$

where *σ_f_* is the stress in the film, *E*_0_ *t*_0_, and *v*_0_ are the elastic modulus, thickness, and Poisson’s substrate ratio, respectively. Δ*k* is the curvature change of the film-substrate system. The substrate curvature change *k_i_* caused by film mismatch strain is given in reference [24]:

(2) $$k_{i}=-{\frac{\sum_{j=1}^{i}B_{j}\varepsilon_{j}^{m}\left\{\frac{1}{2}\left(Z_{j+1}^{2}-Z_{j}^{2}\right)-S(Z_{j+1}-Z_{j})\right\}}{\sum_{j=0}^{i}\frac{B_{j}}{3}\left\{{\left(Z_{j+1}-S\right)}^{3}-{\left(Z_{j}-S\right)}^{3}\right\}}}_{}$$

**Figure 3:**
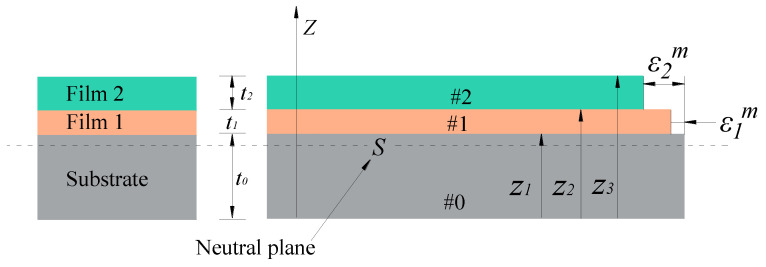
Structural parameters and mismatch strain of double-layer film system on 304SS substrate.

As shown in Figure 3, when depositing thin films, mismatch strain occurs in the film–substrate system due to their different material properties. Severe mismatch strain can lead to film delamination and falling off from the substrate.

*t* is the thickness of the film, *s* is a neutral layer

(3) $$s=t_{0}/2$$

*Z* represents the change in the thickness direction of the film system

(4) $$Z_{i+1}=\sum_{j=0}^{i}t_{j}$$

*B* is a biaxial modulus

(5) Bi=Ei1−vi

where *E* and *v* are the elastic modulus and Poisson’s ratio of the film, respectively. The film stress after loading the deposition temperature mainly depends on the residual thermal stress; the film stress is approximately equal to the thermal stress, and the residual thermal stress is expressed as:

(6) $$\sigma_{t}=\frac{E_{i}}{1-v_{i}}\int_{T_{0}}^{T_{1}}\left(\alpha_{i}-\alpha_{i-1}\right)\;dT\;\left(i=1\ldots n\right)$$

(7) $$\sigma_{t}=\sigma_{f}$$

where *α* is the Coefficient of thermal expansion of the two materials. *T*_1_ and *T*_0_ are deposition temperature and ambient temperature, respectively.

When *i* = 1, the mismatch strain of the transition layer film can be obtained by simultaneous Equations (1)–(7) *ε*_1_*^m^*

(8) $$\varepsilon_{1}^{m}=\frac{2t_{1}\left[B_{0}\left\{{\left(z_{1}-s\right)}^{3}+s\right\}+B_{1}\left\{{\left(z_{2}-s\right)}^{3}+{\left(z_{1}-s\right)}^{3}\right\}\right]}{B_{0}t_{0}^{2}\left\{\frac{1}{2}\left(z_{2}^{2}-z_{1}^{2}\right)-S(z_{2}-z_{1})\right\}}(\alpha_{1}-\alpha_{0})\left(T_{1}-T_{0}\right)$$

When *i* = 2, simultaneous Equations (1)–(7), mismatch strain of insulating film *ε*_2_*^m^*

(9) $$\varepsilon_{2}^{m}=\frac{2t_{2}\left[B_{0}\left\{{\left(z_{1}-s\right)}^{3}+s\right\}+B_{1}\left\{{\left(z_{2}-s\right)}^{3}+{\left(z_{1}-s\right)}^{3}\right\}+B_{2}\left\{{\left(z_{3}-s\right)}^{3}+{\left(z_{2}-s\right)}^{3}\right\}\right]}{B_{0}t_{0}^{2}\left\{\frac{1}{2}\left(z_{3}^{2}-z_{2}^{2}\right)-S(z_{3}-z_{2})\right\}}(\alpha_{2}-\alpha_{1})\left(T_{1}-T_{0}\right)$$

From the Equations (8) and (9) of film mismatch strain *ε*_1_*^m^* and *ε*_2_*^m^*, it can be seen that after the deposition temperature is determined, the degree of film mismatch strain mainly depends on its material properties and thickness. The material properties of the film and substrate are shown in Table 1 [25,26,27,28].

**Figure 4:**
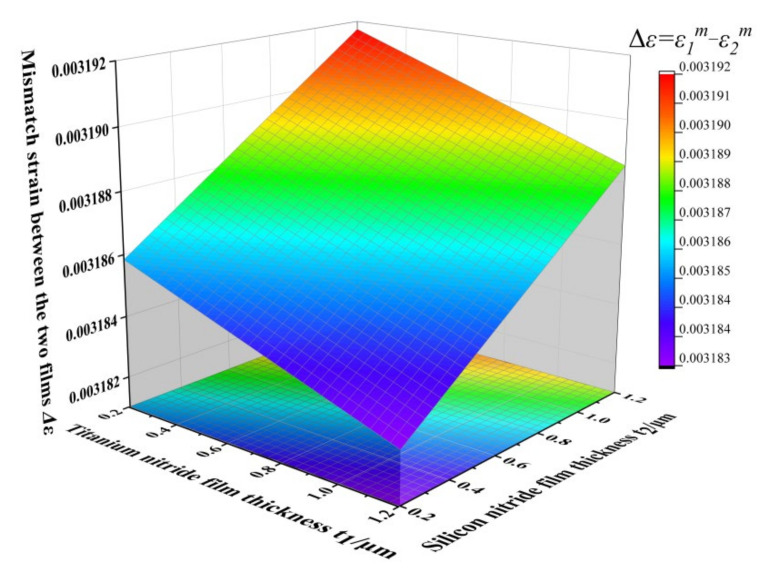
TiN/Si_3_N_4_ film thickness and interlayer mismatch strain Δ*ε*.

To reduce the mismatch strain between the two films, expressed by Δ*ε* = (*ε*_2_*^m^* − *ε*_1_*^m^*), the deformation of the film is reduced, which can prevent the film from tearing due to excessive tensile stress or compressive stress. The relationship between Δ*ε* and film thickness in the TiN/Si_3_N_4_ thin-film system is depicted in Figure 4.

The change of silicon nitride film thickness has a more significant influence on the mismatch strain between the films. Increasing the thickness ratio of titanium nitride film to silicon nitride film can reduce the mismatch strain between the films.

### 2.2. Deposition of Titanium Nitride Film

TiN films were deposited on 304SS substrates using FJL-560a magnetron and ion beam composite sputtering deposition system. Controls the flow of nitrogen (1 sccm, 1.5 sccm, 2 sccm,2.5 sccm, 3 sccm) and argon (50 sccm) into the vacuum chamber. Apply substrate bias voltage (0–−100 V), deposition power 100 W, and deposition pressure 1.2 Pa. Before depositing the film, the substrate is polished to obtain a surface roughness of 30–50 nm; the roughness value is measured by an optical profilometer (Bruker contour GT-k), ultrasonic cleaning with acetone and anhydrous alcohol for 20 min.

### 2.3. Characterization of Titanium Nitride Films

Before analysis, all films were etched by Ar^+^ ions for 90 s to remove contaminants from the surface. The phase constituent of films was measured by an X-ray diffractometer (Bruker D8 Advance), in which the grazing incident mode with Cu Kα was applied. The grazing incidence angle of 0.5° and the scanning range of 30°–50° was set. The scanning speed was 1°/min, and the grazing wavelength was λ = 0.154 nm. The morphology of the TiN film surface of 5 × 5 μm under different negative bias voltages and nitrogen flow rates was measured by an atomic force microscope (Bruker Dimension Icon, Bruker Corporation, Billerica, MA, USA). The local and cross-section morphologies were further studied. The RMS (root mean square) roughness value of the AFM diagram was obtained by discrete approximate calculation.

### 2.4. Preparation of Silicon Nitride Films and Nanoindentation Tests

The silicon nitride film was fabricated by a SI500D Plasma Enhanced Chemical Vapor Deposition System. Process parameters are power 350 W, deposition pressure 4 Pa, deposition temperature 300 °C, NH_3_ flow 8 sccm, SiH_4_ flow 145 sccm, argon flow rate 140 sccm, and the RF frequency of film sputtering 13.56 MHz. After sputtering, the film is cooled down to 30 °C in the vacuum chamber and removed.

The nano-hardness and elastic modulus of the TiN/Si_3_N_4_ film were measured by a nanoindenter (Bruker HYSITRON TI980, Bruker Corporation, Billerica, MA, USA). Load and unload rates with tests were fixed at 200 μN/s. The indentation test used a high-load diamond probe produced by Conoshpher, the probe radius was 5 μm, and Young’s modulus 1140 GPa. The values of H and E presented in this paper are the average of three individual indentations. Film thickness was measured using a step meter (KLA-TencorP7). The film thickness was calculated from the average value of the six selected test points, and the film deposition rate was obtained by dividing the film thickness by time.

## 3. Results and Discussions

### 3.1. Effect of Substrate Negative Bias Voltage on Surface Morphology of TiN Films

**Figure 5:**
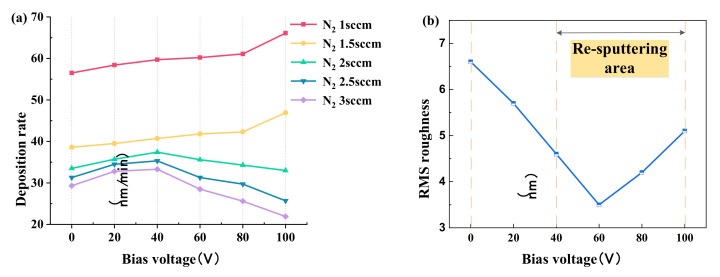
(**a**) Effect of different process parameters on the deposition rate of TiN film (**b**) Variation of RMS roughness of TiN film with negative bias voltage at a nitrogen flow rate of 3 sccm.

The roughness of the film has an important effect on the binding force. Figure 5a shows the rate of TiN film deposition with increasing negative bias voltage at different nitrogen flow rates. The RMS roughness value of the TiN film is given in Figure 5b.

**Figure 6:**
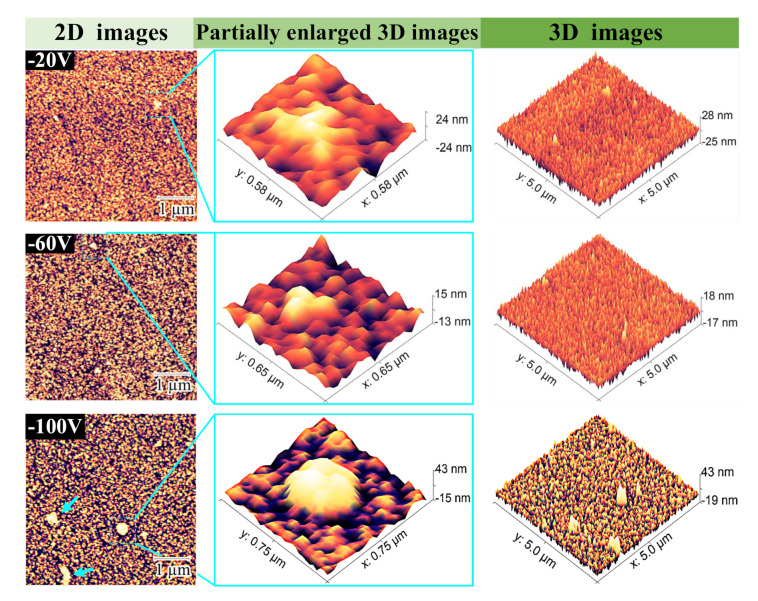
The 2D, 3D, and partially enlarged images of titanium nitride films deposited at substrate bias voltages of −20 V, −60 V, and −100 V.

The evolution of the surface microstructures of the films is shown in the AFM images. Figure 6 shows the surface topography and local three-dimensional images of 5 × 5 μm at different negative bias voltages.

In Figure 5b and Figure 6, it can be observed that when the bias voltage increases from 0 V to −60 V, the film surface appears smooth. According to the partially enlarged 3D images, the granular bulge structure became lower, and the RMS roughness decreased from 6.6 nm to 3.5 nm. However, as the bias voltage increases to −100 V, more and larger granular bulge structures appear on the surface of the film, marked by arrows in the 2D image. The roughness increases to a relatively high 5.1 nm.

The increase of negative bias voltage will lead to the re-sputtering effect of TiN film, which can be identified by the change of film deposition rate in Figure 5a. When the nitrogen flow rate is greater than 2 sccm, the re-sputtering effect occurs at −40 V, resulting in a decrease in film thickness and deposition rate. When the bias voltage is −60 V, the film roughness reaches optimum; the increased negative bias leads to relatively higher bombardment energy, and additional incident energy makes Ti and N combine to form dense nanocrystalline structures—the key to the formation of TiN phase, discussed below.

### 3.2. Effect of Nitrogen Gas Flow on Phase Composition and Microstructure of TiN

**Figure 7:**
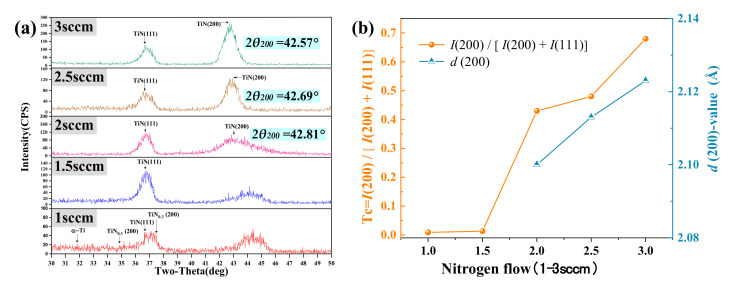
(**a**) The XRD image and phase composition of the TiN film are obtained at a bias of −60 V and a nitrogen flow rate of 1–3 sccm. (**b**)Effect of Nitrogen Flow on TiN(200) Peak Orientation and lattice plane distance (*d*_200_).

The phase composition of TiN film is significantly affected by the change of nitrogen flow rate. Figure 7 shows the XRD patterns of titanium nitride samples under different nitrogen flow rates when the bias voltage is −60 V.

The phase composition of the film sample with a nitrogen flow of 1sccm is different from that of other film samples. Due to the lack of TiN phase, the film consists of TiN(111), TiN_0.3_(200) phase, and a small amount of α-Ti phase, which should be attributed to the severe nitrogen deficiency in the film. The peak value of TiN(111) can be observed in the films deposited at 1.5 sccm nitrogen flow, but no significant TiN(200) peak orientation was observed. However, with the increase of nitrogen flow (2–3 sccm), the mixed-phase of TiN(111) and (200) appears.

The preferred orientation of TiN(200) is quantified by the texture coefficients (*Tc*) = *I*(200)/[*I*(111) + *I*(200)], as shown in Figure 7b, where *I* is the integral intensity of the corresponding Bragg peak in the XRD image. The lattice plane distance (*d*_200_) is evaluated using the standard Bragg’s relation, as shown in equation (10):

(10) d200=nλ2sinθ200

where θ_200_ is the diffraction angle in Figure 7a and *λ* is the X-ray wavelength (1.54Å). As shown in Figure 7b, the *Tc* value increases with the increase of nitrogen flow, and the preferential orientation of the TiN(200) peak increases. When the nitrogen flow reaches 2–3sccm, the lattice plane distance (*d*_200_) increases continuously due to the slight angle offset of TiN (200).

**Figure 8:**
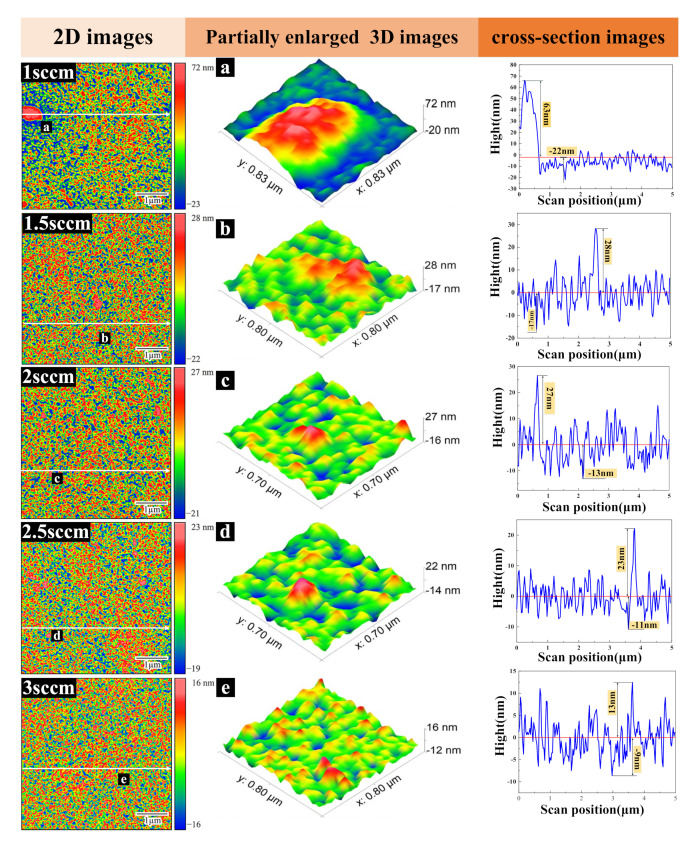
When the bias voltage is −60 V, and the nitrogen flow rate is 1–3 sccm, the 2D images, 3D local partially enlarged images (**a**–**e**), and cross-sectional images of titanium nitride film.

Qi Runze et al. [29,30] showed that the samples with a clear TiN(200) orientation had higher film smoothness than the samples with TiN(111) orientation, which is also confirmed by experiments and shown in Figure 8.

Observing the partially enlarged 3D image in Figure 8, it can be seen that in the sample with a nitrogen flow rate of 1sccm, the granular bulge structure is clearly visible; however, with the increase of nitrogen ratio in the working gas, the bulge characteristics of the film decrease significantly, which is due to the disappearance of the large-grained α-Ti phase. From the 2D image, it could be concluded that with the further increase of nitrogen ratio to 3sccm, a smoother area appears on the sample surface. The cross-section image suggests that the peak-to-peak (PP) and peak-to-valley (PV) ratios decrease from (63 nm, −22 nm) to (13 nm, −9 nm) respectively, indicating that the change of TiN(200) peak intensity caused by nitrogen flow has a significant effect on the film smoothness.

### 3.3. Film Thickness and Nano Indentation Experiment

**Figure 9:**
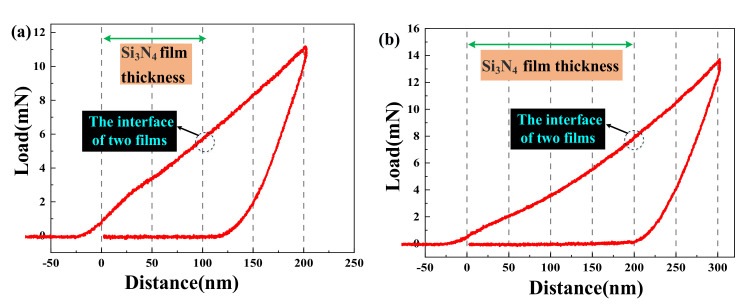
Load displacement curves of two different maximum indentations of TiN/Si_3_N_4_ films: (**a**) 203 nm, (**b**) 302 nm.

Ultrahardness at the interface between TiN and Si_3_N_4_ films significantly improves the hardness of the composites. In this paper, the maximum depth of indentation is slightly greater than the thickness of silicon nitride film so as to avoid affecting the accuracy of results due to too shallow indentation. The loading displacement curves of two silicon nitride films with different thicknesses are given in Figure 9.

For the purpose of the study, the silicon nitride film thickness of samples 1–3 were 100 nm, the titanium nitride film thicknesses were 800 nm, 600 nm, and 400 nm, and the maximum indentation depth was 203.1 nm, as shown in Figure 9a. The silicon nitride film thickness of samples 4–6 was 200 nm, the titanium nitride film thicknesses were 800 nm, 600 nm, and 400 nm, and the maximum indentation depth was 302.7 nm, as shown in Figure 9b.

**Figure 10:**
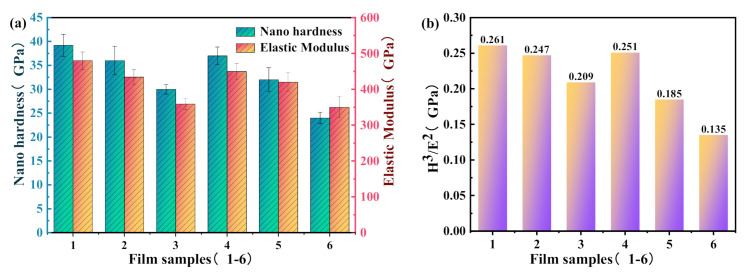
Mechanical properties of TiN/Si_3_N_4_ film: (**a**) nano-hardness and elastic modulus. (**b**) H^3^/E^2^ ratio.

The indentation data such as nanohardness and elastic modulus of TiN/Si_3_N_4_ film are given in Figure 10a, and in order to qualitatively evaluate the toughness of the film, the ratio of H^3^/E^2^ is calculated, as shown in Figure 10b.

It is observed that sample 1 shows the highest nanohardness (H) of 39.2 GPa and has a relatively high H^3^/E^2^ of 0.261 GPa. The elevated hardness of sample 1 proves that there is lattice hardening at the interface. The sample also has good insulation properties. With the decrease of titanium nitride and film thickness, and with the increase of silicon nitride film thickness, the nanohardness and toughness of the sample gradually decrease, as shown in Figure 10. The nanohardness and toughness of sample 6 were reduced to 26 GPa and 0.135 GPa. Veprek et al. [31,32,33,34] pointed out that thicker SiN_x_ could weaken the Ti–N bond near the interface, destroy the coherent interface, and decrease the hardness of the film.

**Figure 11:**
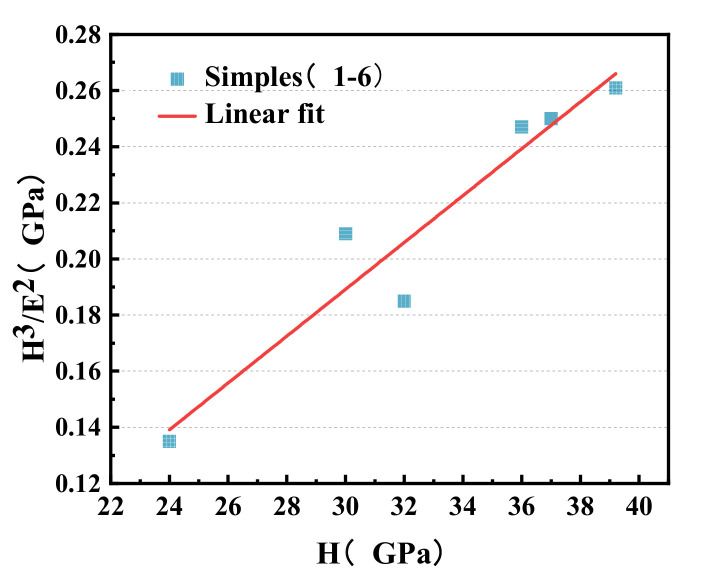
Dependence of toughness H^3^/E^2^ on hardness H.

In order to establish a TiN/Si_3_N_4_ film system with both hardness and toughness, the relationship between toughness (H^3^/E^2^) and hardness (H) is described, and a linear fit is generated, as shown in Figure 11.

It can be seen from the fitting line that TiN/Si_3_N_4_ film with greater hardness usually has a higher H^3^/E^2^ ratio.

**Figure 12:**
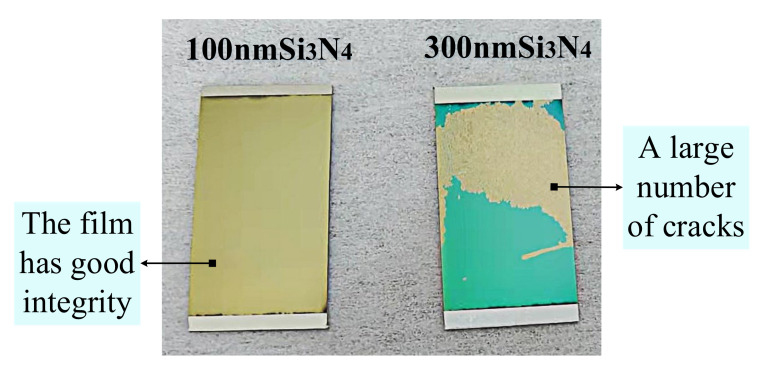
Si_3_N_4_ films with different thicknesses deposited on TiN films with the same thickness: 100 nm (**left**), 300 nm (**right**).

In conclusion, increasing the ratio of titanium nitride film thickness to silicon nitride film thickness can improve the nanohardness and toughness of TiN/Si_3_N_4_ film, which indicates the feasibility of reducing the mismatch strain between the two films and improving the film properties. In subsequent experiments, the thickness of silicon nitride film was further increased to 300 nm. After sputtering, a large number of cracks appeared in the silicon nitride film placed in the air, as shown in Figure 12.

It can be observed that the surface of the single-layer 300 nm silicon nitride film sample is cracked, and the integrity of the sample is seriously compromised. This result is in sharp contrast to that of the single-layer 100 nm silicon nitride film sample. The failure of TiN/Si3N4 film is likely due to the introduction of a large amount of thermal stress during the deposition of silicon nitride film, resulting in excessive mismatch strain between silicon nitride film and titanium nitride film. This strain caused the film cracking, which is also one of the main reasons for reducing film toughness.

## 4. Conclusions

(1) The surface morphology and root mean square roughness of titanium nitride films under substrate negative bias voltage (0~−100 V) were revealed by atomic force microscope. The smoothest sample was obtained at −60 V bias and 3 sccm nitrogen flow, with an RMS of 3.5 nm. Then, with the increase of negative bias voltage, the surface smoothness of the film decreases, and the RMS increases to 5.1 nm at −100 V.
(2) XRD analysis was carried out on the film (−60 V) with optimal smoothness. When the nitrogen flow rate is sufficient, the sample has a mixed phase of TiN(111) and (200). With the increase of nitrogen flow rate, the intensity of the TiN(200) phase increases continuously. It is observed by AFM that the smoothness of film samples becomes higher with the increase of TiN(200) peak orientation.
(3) A TiN/Si_3_N_4_ functional film system for cutting force measurement is established. In the nanoindentation experiment, the combination of 800 nm titanium nitride and 100 nm silicon nitride film showed the optimal hardness of 39.2 GPa, H^3^/E^2^ ratio of 0.261 GPa, and sound insulation performance. In high-speed machining, high enough hardness and toughness is not only the premise of accurate data acquisition but also a guarantee of service longevity.
(4) TiN/Si_3_N_4_ films with greater hardness usually have a higher H^3^/E^2^ ratio. The subsequent increase in the thickness of silicon nitride film to 300 nm led to film cracking and sample failure. These observations, combined with the results of the nanoindentation experiment, verify our theoretical mismatch film-strain analysis.

## Figures and Tables

**Table 1:** Material properties of TiN film, Si_3_N_4_ film, and 304SS substrate.

Material	Elastic Modulus (GPa)	Poisson’s Ratio	Thermal Expansion Coefficient (10^−6^/K)
304SS	195	0.25	17.2
TiN Film1	279	0.25	7.4
Si_3_N_4_ Film2	304	0.24	2.45
